# Fusobacterium in oral bacterial flora relates with asymptomatic brain lesions

**DOI:** 10.1016/j.heliyon.2024.e39277

**Published:** 2024-10-12

**Authors:** Yoshie Kato, Masahiro Takamura, Koichiro Wada, Haruki Usuda, Satoshi Abe, Shingo Mitaki, Atsushi Nagai

**Affiliations:** a Department of Neurology, Shimane University Faculty of Medicine, Japan; b Department of Pharmacology, Shimane University Faculty of Medicine, Japan

**Keywords:** Oral bacterial flora, Asymptomatic brain lesions, Risk factor, *Fusobacterium nucleatum*

## Abstract

**Background:**

Specific bacterial species in the oral cavity contribute to cerebral hemorrhage and microbleeds. The relationship between oral bacterial flora and asymptomatic brain lesions (ABL) remains unclear. This study aimed to investigate this relationship in a healthy Japanese cohort.

**Methods:**

This cross-sectional study included participants who underwent health examinations at our Brain Dock facility between October 2020 and March 2021. The oral microbiomes of participants with and without ABL were compared using magnetic resonance imaging. To extensively assess the oral bacterial flora, the differences in genes and species compositions between the ABL and noBL (without brain lesions) groups were statistically evaluated via extensive analysis using 16S rRNA gene-based cloning.

**Results:**

Among 143 patients, 48.3 % had ABL. In the univariate analyses, *Fusobacterium* and *Leptotrichia* were associated with ABL (P = 0.017 and P < 0.001, respectively). In the adjusted models, *Fusobacterium* was associated with ABL (P = 0.006). In an intergroup comparison of seven *Fusobacterium* species, *F. nucleatum*, *F. naviforme*, and *F. canifelinum* were associated with ABL (P < 0.001, P = 0.002, P < 0.001).

**Conclusions:**

The elevation of *Fusobacterium* in the ABL indicates the importance of the microbiome in the oral cavity as a factor in inducing cerebral small-vessel disease in healthy individuals, whose preventive approach might have an impact on therapeutic applications.

## 1 Introduction

Growing evidence has shown that the human microbiome is important for maintaining human health [1]. Microbiota constituents in the oral cavity play a role in the regulation of metabolic, pro-inflammatory, and immune functions in the whole body, and these alterations are thought to affect the induction of systemic disease conditions, such as dyslipidemia, diabetes mellitus, hypertension, peripheral artery diseases [2], and cardiovascular diseases, causing an increased risk of all-cause mortality [3]. Because ordinal tooth brushing, dental extraction, and periodontitis cause bacteria to enter the bloodstream and release cytotoxins and pro-inflammatory factors, changes in the microbiome may affect the blood-brain barrier condition or lead to the destruction of the vessels and parenchyma in the brain. Epidemiological studies have found that periodontal disease is a potential risk factor for cerebrovascular accidents independent of vascular risk factors [4,5]. Specific type of bacteria, collagen-binding protein of *Streptococcus mutans*, was also suggested to be associated with intracerebral hemorrhage and deep microbleeds in vivo and a hospital cohort study [6,7].

The oral microbiota consists of several hundred bacterial species [8], and it is constructed by complex components such as the teeth, gingiva, and tongue [9]. As periodontal inflammation alters the content and balance, the chronic state of periodontitis may change homeostasis and lead to the development of the disease condition by affecting its profile. Periodontitis is recognized as a common disease affecting more than 40 % of adults [10,11], causing a big impact on disease occurrence. The management of this condition is important to prevent various diseases.

Pathogenic species do not usually play a role in the periodontal microbiota as a single pathogenic entity [12] and that antagonistic and/or synergistic relationships among several species determine the pathogenic role of the microbiota. Wide-range analysis using 16S rRNA gene-based cloning has been developed to extensively detect bacterial composition and confirm the relationship between the oral microbiome and diseases such as colorectal tumors, head and neck squamous cell cancer, and migraine [[13], [14], [15]]. Since whether changes in oral microbiome affect the occurrence of cerebrovascular disease remains unclear, we investigated the relationship between the oral microbiome and the incidence of asymptomatic brain lesions (ABL) as markers of cerebral small-vessel disease in a Japanese cohort who underwent health checkup using extensive gene-based cloning for the health maintenance of healthy individuals.

## 2 Methods

### 2.1 Study population

**Fig. 1:**
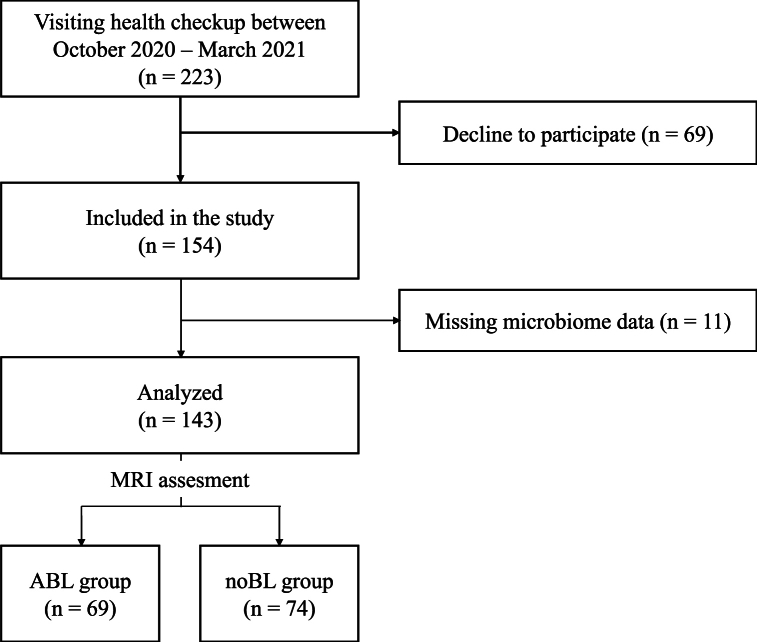
Flow diagram of study participants. ABL, asymptomatic brain lesions; noBL, without brain lesions.

Between October 2020 and March 2021, 223 Japanese individuals underwent a health checkup at the Health Science Center in Shimane, Japan. Of these, 154 participated in this cross-sectional study, and 69 did not agree to participate because of the time and effort required to collect saliva samples. Medical history taking, neurological examinations, head magnetic resonance imaging (MRI), blood tests, and saliva sampling were performed for oral microbiome assessment. After assessment, 11 participants were excluded because of missing microbiome data. Finally, 143 participants (88 men and 55 women), aged 35–85 years (mean 64.4 ± 11.8 years), were analyzed (Fig. 1). The participants had one or more teeth, had not gargled or brushed for more than 2 h, and had not taken antibiotics or anti-inflammatory drugs, including steroids, within 1 month.

This study was approved by the Ethics Committee of the Health Science Center Shimane and Shimane University School of Medicine in Japan (Permission no. 4696). All participants provided written informed consent prior to participation. This study was conducted in accordance with the Declaration of Helsinki.

### 2.2 Assessment of background characteristics

Age, sex, blood pressure, pulse rate, medical history, smoking and drinking status, and medication use were recorded. Trained neurologists and certified clinical psychologists performed neurological examinations, including the Mini-Mental State Examination (MMSE) and Frontal Assessment Battery (FAB). Hypertension was defined as a blood pressure ≥140/90 mmHg or antihypertensive drug use. Serum total cholesterol (TC), high-density lipoprotein cholesterol (HDL-C), low-density lipoprotein cholesterol (LDL-C), triglyceride (TG), and fasting plasma glucose levels were measured. Hyperlipidemia was defined as a serum cholesterol level ≥220 mg/dL or the use of lipid-lowering drugs. Diabetes was defined as a fasting blood glucose level ≥126 mg/dL, random blood glucose level ≥200 mg/dL, hemoglobin A1c (HbA1c) (NGSP) ≥ 6.5 %, or antidiabetic drug use. Chronic kidney disease (CKD) was defined as a glomerular filtration rate (GFR) < 60 mL/min. Smoking and drinking status were defined as follows: a smoker who habitually smoked at least one cigarette per day and a drinker who consumed ≥180 mL of alcohol per day.

### 2.3 Assessment of brain imaging data

Magnetic resonance imaging (MRI) was performed using a Philips Ingenia CX Dual 3.0-T scanner at the Health Science Center, Shimane. The entire head of each patient was scanned using T2-weighted imaging (T2WI), T1-weighted imaging (T1WI), fluid-attenuated inversion recovery imaging (FLAIR), and gradient-echo T2∗WI with a slice thickness of 5 mm. An experienced radiologist diagnosed asymptomatic brain lesions using MRI. Cerebral microbleeds (CMBs) were defined as homogeneous round foci of signal loss with diameters of 2–10 mm on T2∗WI. Periventricular hyperintensities (PVH) and deep and subcortical white matter hyperintensities (DSWMH) were evaluated based on their distinct subcortical distributions on FLAIR images. PVH and DSWMH were assessed using the Fazekas rating scale [16] (0–3 for PVH and 0–3 for DSWMH). For statistical purposes, the PVH and DSWMH grades were dichotomized. PVH grades 0–1 were defined as PVH- and grades 2–3 as PVH+. Similarly, DSWMH grades 0–1 were defined as DSWMH- and grades 2–3 as DSWMH+. All MRI findings were read and determined separately by an experienced neurologist and a neuroradiologist. Based on MRI assessment, participants who exhibited CMBs (n = 21, 14.7 %), PVH+ (n = 16, 11.2 %), DSWMH+ (n = 44, 30.8 %), or silent brain infarcts (n = 15, 10.5 %) were assigned to the ABL group.

### 2.4 Sample preparation and bacterial analysis

The participants washed their mouths with 1 mL distilled water for 30 s. The fluid was collected as a bacterial sample. Bacterial DNA was extracted from each sample using a QIAamp DNA Microbiome Kit (QIAGEN) according to the manufacturer's instructions. Bacterial DNA was stored at −80 °C until use. Streptococcus mutans (S.m) and S.m-derived collagen-binding protein gene (cnm) were detected by polymerase chain reaction (PCR) using specific primer pairs and electrophoresis. The primer sequences were as follows: S.m: forward 5′-GGCACCACAACATTGGGAAGCTCAGTT-3′, reverse 5′-GGAATGGCCGCTAAGTCAACAGGAT-3' (product size, 433 bp) [17]; cnm: forward 5′-CTGAGGTTACTGTCGTTAAA-3′, reverse 5′-CACTGTCTACATAAGCATTC-3′ [18]. In addition, we comprehensively analyzed bacterial sequences using a new-generation sequencing technique. Briefly, the genome sequences of the V3-V4 region of the bacterial 16S rRNA were amplified by PCR. The estimated length of the target region was approximately 600 bp. After purifying the amplicon with AMPure XP beads (BECKMAN COULTER), barcode sequences were added to the amplicons to label the samples using the Illumina Nextera XT Index kit v2 (Illumina. K.K., Japan). The purified samples were diluted to 4 nmol/L using 10 mmol/L Tris-HCl (pH 8.0) and the same volume of each sample was pooled for multiplex sequencing. The multiplexed library pool (6 pmol/L) was spiked with 5 % phiX control DNA (6 pmol/L) and sequenced using a 2 × 300-bp paired-end run on a MiSeq platform using the MiSeq Reagent Kit v3 (Illumina). Bacterial annotation and calculation of the number of reads was performed using the 16S metagenomics application (Illumina).

### 2.5 Statistical analysis

In this study, the relationship between the oral microbiome and incidence of asymptomatic brain lesions was examined in two stages. First, genus-level differences between the groups were explored to capture robust intermediate-level microbiome changes related to ABL. Next, based on the results of the genus analysis, a targeted species analysis was conducted to understand oral microbiome changes in detail.

In the first stage, wide-range analysis, background information, and genus-level values were compared between groups using univariate analysis. For continuous variables, the Mann-Whitney *U* test was used; for categorical variables, Chi-squared (χ^2^) test was used. These analyses exploited the possible genus changes and confounding variables. Next, a multivariate logistic regression analysis with the forced entry method was conducted to determine the survival of the genus change after controlling for the effects of other variables. Finally, in the second stage, detailed analysis, the species specified by genus analysis were tested by group comparisons using the Mann-Whitney *U* test. In addition, as a supplemental analysis, we created subgroups matched for age gender and re-examined the univariate analysis. Matching was performed by ‘MatchIt’ package in R using exact matching method. All statistical analyses were conducted using JASP (version 0.16), and the significance level was set at P < 0.05.

## 3 Results

### 3.1 Baseline characteristics

Based on the brain assessment, the participants were divided into two groups: 69 in the ABL group and 74 in the without brain lesion (noBL) group. Baseline characteristics and group comparison results are presented in Table 1. Age and hyperlipidemia ratios were significantly higher in the ABL group.

**Table 1:** Baseline clinical chareacteristics of participants.

	All participants			
		ABL	noBL	*P* value
	(n = 143)	(n = 69)	(n = 74)	
Age (years), mean ± SD	64.4 ± 11.8	69.4 ± 8.7	59.8 ± 12.4	<0.001
Sex, male/female	88/55	43/26	45/26	0.853
Hypertension, n (%)	47 (32.9)	20 (29.0)	27 (36.5)	0.340
Diabetes mellitus, n (%)	25 (17.5)	16 (23.2)	9 (12.2)	0.083
Hyperlipidemia, n (%)	81 (56.6)	32 (46.4)	49 (66.2)	0.017
CKD, n (%)	21 (14.7)	13 (18.8)	8 (10.8)	0.175
Smoking status, n of smoker (%)	28 (19.6)	15 (21.7)	13 (17.6)	0.530
Drinking status, n of drinker (%)	43 (30.1)	22 (31.9)	21 (28.4)	0.648
MMSE, median (IQR)	30 (2.0)	29 (2.0)	30 (1.0)	0.305
FAB, median (IQR)	17 (2.0)	16 (3.0)	17 (2.0)	0.664

ABL, asymptomatic brain lesions; noBL, without brain lesions; CKD, chronic kidney disease; MMSE, Mini-Mental State Examination; FAB, Frontal Assessment Battery; SD, standard deviation; IQR, interquartile range.

**Fig. 2:**
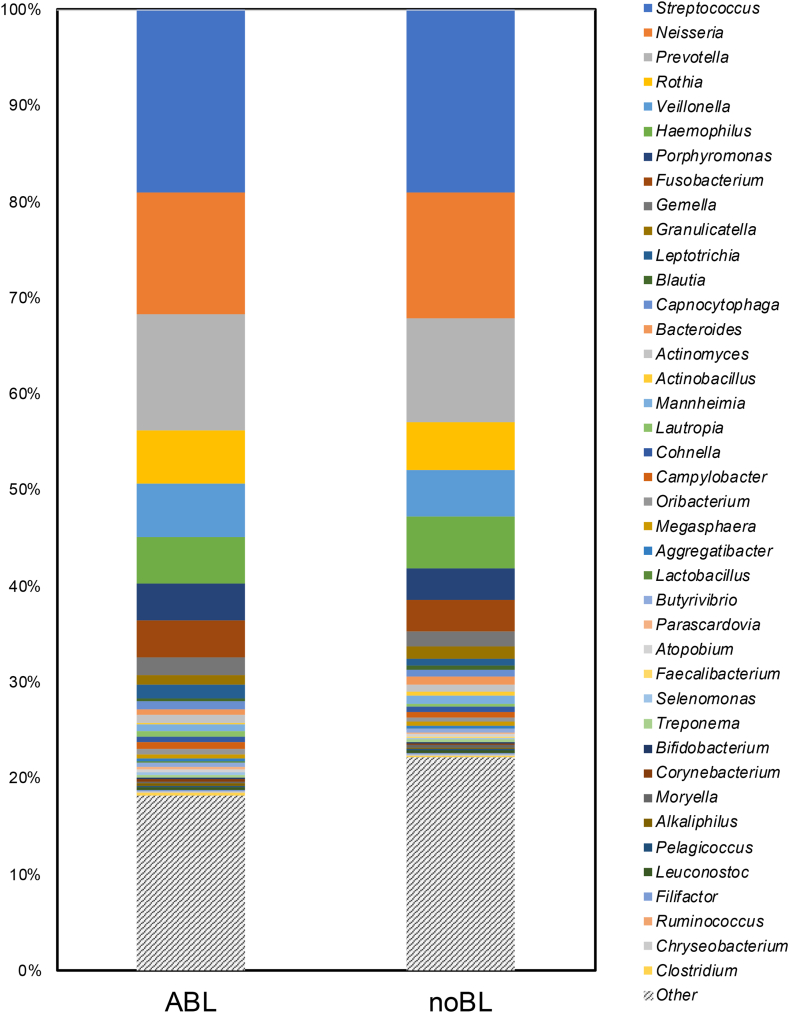
Genus composition of the oral microbiome in the two groups. ABL, asymptomatic brain lesions; noBL, without brain lesions.

The genus composition of the oral microbiome in the two groups were shown as Fig. 2. The most abundant bacterial genera in both groups were *Streptococcus*, *Neisseria*, and *Prevotella*, in that order, which corresponded to typical oral bacteria (Fig. 2). Eleven genera exhibiting moderate composition rates (median of all participants >1 %) were selected and used in subsequent analyses (Table 2 and Fig. 2). As a result of the group comparison, *Fusobacterium* and *Leptotrichia* were significantly higher in the ABL group.

**Table 2:** Exploratory univariate analysis for genus.

Genus	ABL	noBL	*P* value	Effect size
	(n = 69)	(n = 74)		
*Streptococcus*	19.07 (6.40)	18.97 (8.71)	0.864	−0.017
*Neisseria*	12.65 (8.60)	13.16 (11.49)	0.664	0.042
*Prevotella*	12.01 (5.53)	10.79 (8.74)	0.380	0.085
*Rothia*	5.56 (3.52)	4.95 (3.47)	0.437	0.076
*Veillonella*	5.64 (3.30)	4.92 (3.68)	0.280	0.105
*Haemophilus*	4.82 (3.98)	5.42 (3.46)	0.138	−0.144
*Porphyromonas*	3.77 (2.52)	3.27 (3.04)	0.283	0.104
*Fusobacterium*	3.92 (2.78)	3.24 (2.32)	0.017	0.231
*Gemella*	1.78 (1.04)	1.52 (1.40)	0.148	0.141
*Granulicatella*	1.05 (0.72)	1.24 (0.74)	0.164	−0.135
*Leptotrichia*	1.39 (0.80)	0.84 (1.02)	<0.001	0.372

Values are presented as median (interquartile range). ABL, asymptomatic brain lesions; noBL, without brain lesions. The effect size is given according to the rank-biserial correlation.

### 3.2 Association between ABL and microbiome

Multiple logistic regression analysis was conducted with ABL as the dependent variable (ABL group vs. noBL group). The independent variables were age, hyperlipidemia, *Fusobacterium*, and *Leptotrichia*. The results showed significant effects of age and *Fusobacterium*. However, the effect of *Leptotrichia* was not statistically significant (Table 3).

**Table 3:** Multiple logistic regression analysis of selected variables.

Variables				Wald Test				95 % Confidence Interval	
	Estimate	Std. Error	z	Wald Statistic	df	P value	Odds Ratio	Lower	Upper
(Intercept)	−6.774	1.539	−4.402	13.379	1	<0.001	0.001	0.000	0.023
Fusobacterium	0.317	0.115	2.759	7.613	1	0.006	1.374	1.096	1.721
Leptotrichia	0.267	0.224	1.192	1.421	1	0.233	1.305	0.842	2.023
Age	0.085	0.021	4.126	17.026	1	<0.001	1.089	1.046	1.134
Hyperlipidemia	−0.753	0.394	−1.910	3.650	1	0.056	0.471	0.218	1.020

### 3.3 Species analysis

Seven *Fusobacterium* spp. were detected in the samples (Table 4). Group comparisons of each species revealed a significant elevation in *F. nucleatum*, *F. naviforme*, and *F. canifelinum* in the ABL group compared to the noBL group.

**Table 4:** Targeted univariate analysis for species of *Fusobacterium*.

Species	ABL	noBL	*P* value	Effect size
	(n = 69)	(n = 74)		
*F. periodonticum*	1.38 (0.90)	1.34 (1.31)	0.604	0.051
*F. nucleatum*	0.65 (0.74)	0.38 (0.43)	<0.001	0.333
*F. naviforme*	0.55 (0.47)	0.41 (0.31)	0.002	0.307
*F. gonidiaformans*	0.03 (0.03)	0.03 (0.03)	0.960	0.005
*F. simiae*	0.01 (0.02)	0.01 (0.01)	0.086	0.167
*F. canifelinum*	0.01 (0.01)	0.00 (0.00)	<0.001	0.337
*F. necrophorum*	0.00 (0.00)	0.00 (0.00)	0.354	0.230

Values are presented as median (interquartile range). ABL, asymptomatic brain lesions; noBL, without brain lesions; *F*, *Fusobacterium*. The effect size is given according to the rank-biserial correlation.

### 3.4 Supplemental analysis

To evaluate the robustness of the elevation of *Fusobacterium* in ABL group, we conducted a analysis that reduced the effects of age with matching. When comparing the age-and-sex matched ABL group (n = 31) and the noBL group (n = 36), only the difference in *F. nucleatum* was remain significant (Supplemental Table 1). In addition, we conducted the exploratory univariate analysis again for the eleven genera (Table 2) using the age-and-sex matched dataset. No significant group differences were observed (Supplemental Table 2).

## 4 Discussion

In this study, we performed an oral bacterial flora analysis using a 16S rRNA gene-based cloning method combined with our Japanese health examination cohort, which has long been established to investigate the risk factors for stroke and dementia [[19], [20], [21], [22], [23]]. Univariate analysis of the genus revealed that *Fusobacterium* and *Leptotrichia* were associated with ABL, and multivariate logistic regression analysis showed that the effect of *Fusobacterium* was significant after controlling for other factors. Furthermore, univariate analysis of species focused on *Fusobacterium* revealed that *F. nucleatum*, *F. naviform*, and *F. canifelinum* were significantly more abundant. The present study is the first to show a relationship between these bacteria and the occurrence of cerebral small-vessel disease.

Our study, focusing on ABL, included the clinical importance based on several pieces of evidence. First, the presence of ABL increases the risk of subsequent stroke, independent of cardiovascular risk factors. Vermeer et al. [24] in a study of 1077 individuals from the Rotterdam Scan Study has reported that the presence of silent brain infarcts and white matter lesions increased the risk of stroke 4-fold, independent of other stroke risk factors. The meta-analysis including eight studies (10,427 participants) revealed that silent brain infarct is an independent predictor of stroke incidence, with a hazard ratio (HR) of 2 [25]. Similarly, our cohort study demonstrated that the presence of CMBs was a risk factor for both future ischemic and hemorrhagic stroke with an HR of 4.5 and 50, respectively [20]. Second, the presence of ABL is associated with cognitive impairment and an increased risk of dementia. Several meta-analyses of cross-sectional studies have shown that silent brain infarction, white matter hyperintensities, and CMBs are associated with cognitive dysfunction [[26], [27], [28]]. Furthermore, Debette et al. [29] in a study of 1694 participants from the Framingham Offspring Study revealed that the presence of silent brain infarcts increased the risk of dementia with an HR of 6, and a meta-analysis including nine longitudinal studies demonstrated that the presence of white matter intensities was also associated with an increased risk of dementia [30]. Considering this evidence, the present study includes the significance of the predictors of stroke and dementia because specific changes in the oral microbiome were related to ABL, which is ‘clinically silent’ at the time of detection.

Periodontitis has been known to be associated with the incidence of cardioembolic and thrombotic stroke subtypes [31,32]. However, previous nationwide studies showing the relationship between periodontitis and stroke used only interviews or hospital records to analyze data on dental care and stroke occurrence. The detection of a specific type of microbiome or changes in microbiome construction may contribute to the prevention of cerebrovascular disease occurrence. Our study revealed that several genera of *fusobacterium*, which are ubiquitous gram-negative anaerobic bacteria in the oral cavity, had a higher ratio in the population with ABL. Since *Fusobacterium* species are known to constitute the oral microbiome and are associated with periodontitis [33], the bacteremia or inflammatory environment induced by them may affect vascular factors of small-vessel disease derived from arteriosclerosis, as suggested in the pathophysiology of diseases such as carcinogenesis, Alzheimer's disease, and multiple sclerosis [[34], [35], [36]]. *F. nucleatum* can activate macrophage PI3K-AKT/MAPK/NF-κB signal pathways, promote inflammation, enhance cholesterol uptake, reduce lipid excretion, and promote lipid deposition, which may be one of its main strategies promoting the development of arteriosclerosis and atherosclerosis [37].

*Fusobacterium* are strictly anaerobic microorganisms ubiquitous in the oral cavity, and *F. nucleatum* cause a wide variety of human infections, commonly seen in periodontitis, and are one of the main protagonists of abscesses in many organs, including the brain [38]. It was found to be the cause of brain abscesses, often after cranial or dental surgery, indicating that it passes through the blood-brain barrier [39]. This mechanism involves the function of the surface adhesin FadA in adhesion and invasion of host cells, including epithelial and endothelial cells [40,41], eliciting a variety of host inflammatory responses [42]. In general, known fusobacterial adhesins (Aid1, CmpA, Fap2, FomA, FadA, and RadD) play a vital role in microbial co-aggregation, mediating invasion, and facilitating the spread of bacteria [43]. A recent report showed that detection of antibodies against *F. nucleatum* remained an independent predictor of unfavorable outcomes in a follow-up study of acute stroke patients [44]. Although only *F. nucleatum* has been documented in the literature, we found three candidate *Fusobacterium* species that may be involved in the occurrence of ABL. *F*. *naviforme* strain showed 93 % sequence similarity to *F. nucleatum* subspecies in the 16S-23S rDNA internal transcribed spacer sequence [45]. *F. naviforme* is associated with aggressive periodontitis [46]. *F. nucleatum/naviforme* cause severe liver abscesses [47], and a similar synergistic phenomenon might also induce a severe case of concurrent infection in the brain. Robust microbiome community changes are thought to occur when moving from healthy to diseased states. *F. caniferinum* is thought to belong to a distinct species closely related to *F. nucleatum* [48] and has been detected in the oral cavity of dogs and cats [48,49]. It is likely that its detection has become more common with the recent pet boom; however, attention should be paid to the involvement of this species in future studies.

Taken together, our cross-sectional research indicated a relationship between oral *Fusobacterium* and silent small-vessel changes in the brain MRI of a healthy cohort for the first time. These results suggest the need to focus on changes in the oral microbiome as a mechanism for cerebrovascular events.

### 4.1 Limitation

This study has several limitations. First, the cross-sectional study design limits causal inferences. Second, the participants were recruited from a health examination cohort, which may not accurately represent the entire population of Japan. Third, the univariate analyses conducted in this study were not corrected for family wise errors due to the explanatory purpose of this study. It is also important to note that many of the significant differences between groups disappeared in the supplementary analysis using age-and-sex matching. This result may have been limited in power by the reduction in sample size due to matching. Nevertheless, significant difference remained among *Fusobacterium* species, suggesting the significance of additional validation in the future. Further replications in different cohorts with larger sample size are required to clarify the exact relationship between variables and to confirm these findings. Finally, we could not exclude the possibility of residual confounding by unmeasured determinants, such as medication, diet, or physical activity, which could affect the oral flora content.

## 5 Conclusion

It is worth noting that the specific species of bacteria analyzed among the whole oral microbiome have been shown to independently contribute to asymptomatic brain ischemic factors in our cross-sectional cohort study. Proposing a therapeutic approach to these bacteria would reduce the incidence of cerebrovascular accidents in future studies.

## CRediT authorship contribution statement

**Yoshie Kato:** Writing – review & editing, Writing – original draft, Visualization, Validation, Supervision, Software, Resources, Project administration, Methodology, Investigation, Formal analysis, Data curation, Conceptualization. **Masahiro Takamura:** Writing – review & editing, Writing – original draft, Visualization, Validation, Software, Resources, Methodology, Formal analysis, Data curation, Conceptualization. **Koichiro Wada:** Writing – review & editing, Supervision, Resources, Methodology, Data curation, Conceptualization. **Haruki Usuda:** Writing – original draft, Visualization, Validation, Software, Resources, Methodology, Formal analysis, Data curation, Conceptualization. **Satoshi Abe:** Investigation. **Shingo Mitaki:** Writing – review & editing, Writing – original draft, Validation, Resources, Methodology, Investigation, Formal analysis, Data curation, Conceptualization. **Atsushi Nagai:** Writing – review & editing, Writing – original draft, Visualization, Validation, Supervision, Software, Resources, Project administration, Methodology, Investigation, Funding acquisition, Formal analysis, Data curation, Conceptualization.

## Data availability statement

The raw data associated with this study are available from the corresponding author upon reasonable request.

## Source of Funding

This study was supported by a grant from the 10.13039/100016289Taiju Life Social Welfare Foundation in 2021.

## Declaration of competing interest

The authors declare that they have no known competing financial interests or personal relationships that could have appeared to influence the work reported in this paper.
