# Supplementary material for: Fusobacterium in oral bacterial flora relates with asymptomatic brain lesions
Source: Heliyon. 2024 Oct 12;10(20):e39277. doi: 10.1016/j.heliyon.2024.e39277 (PMC11620239; doi:10.1016/j.heliyon.2024.e39277)
Supplement: Multimedia component 1 [file mmc1.docx]

# Fusobacterium in oral bacterial flora relates with asymptomatic brain lesions

**Supplemental Material**

Supplemental Table 1. Additional analysis of age and sex matched subgroups

|  | ABL | noBL | *P* value | Effect size |
| --- | --- | --- | --- | --- |
| Variables | (n = 31) | (n = 36) |  |  |
| Age (years), mean±SD | 66.5 ±8.4 | 65.6±8.5 | 0.605 | – |
| Sex, male/female | 18 / 13 | 22 / 14 | 0.800 | – |
| Genus |  |  |  |  |
| *Fusobacterium* | 3.92 (2.66) | 3.66 (2.75) | 0.406 | 0.120 |
| Species |  |  |  |  |
| *F. periodonticum* | 1.21 (0.78) | 1.12 (1.72) | 1.000 | 0.000 |
| *F. nucleatum* | 0.71 (1.00) | 0.38 (0.48) | 0.039 | 0.294 |
| *F. naviforme* | 0.55 (0.64) | 0.45 (0.31) | 0.096 | 0.238 |
| *F. gonidiaformans* | 0.03 (0.03) | 0.03 (0.04) | 0.687 | -0.058 |
| *F. simiae* | 0.01 (0.02) | 0.01 (0.01) | 0.529 | 0.091 |
| *F. canifelinum* | 0.01 (0.01) | 0.01 (0.01) | 0.458 | 0.107 |
| *F. necrophorum* | 0.00 (0.00) | 0.00 (0.00) | – | – |

ABL, asymptomatic brain lesions; noBL, without brain lesions. For microbiome data, values are presented as median (interquartile range). The effect size is given according to the rank-biserial correlation.

Supplemental Table 2. Additional exploratory univariate analysis for genus of age and sex matched subgroups

|  | ABL | noBL | *P* value | Effect size |
| --- | --- | --- | --- | --- |
| Genus | (n = 31) | (n = 36) |  |  |
| *Streptococcus* | 19.07 (7.49) | 18.65 (9.24) | 0.769 | -0.043 |
| *Neisseria* | 11.78 (9.25) | 13.37 (10.16) | 0.817 | -0.034 |
| *Prevotella* | 11.03 (4.88) | 10.93 (10.43) | 0.876 | -0.023 |
| *Rothia* | 6.80 (4.06) | 5.52 (4.43) | 0.512 | -0.095 |
| *Veillonella* | 5.40 (2.48) | 5.09 (4.03) | 0.886 | 0.022 |
| *Haemophilus* | 4.43 (3.09) | 5.93 (3.59) | 0.158 | 0.203 |
| *Porphyromonas* | 3.91 (2.03) | 2.84 (3.83) | 0.262 | -0.161 |
| *Fusobacterium* | 3.92 (2.66) | 3.66 (2.75) | 0.406 | -0.12 |
| *Gemella* | 1.75 (1.12) | 1.46 (1.60) | 0.194 | -0.186 |
| *Granulicatella* | 1.16 (0.83) | 1.24 (0.56) | 0.703 | 0.056 |
| *Leptotrichia* | 1.18 (1.06) | 1.07 (1.01) | 0.247 | -0.167 |

Values are presented as median (interquartile range). ABL, asymptomatic brain lesions; noBL, without brain lesions. The effect size is given according to the rank-biserial correlation.
